# Potential Antioxidant Activity of New Tetracyclic and Pentacyclic Nonlinear Phenothiazine Derivatives

**DOI:** 10.1155/2016/9896575

**Published:** 2016-04-04

**Authors:** Godwill Azeh Engwa, Eugene Lekem Ayuk, Benardeth Ujunwa Igbojekwe, Marcellus Unaegbu

**Affiliations:** ^1^Biochemistry, Chemical Sciences Department, Godfrey Okoye University, PMB 01014, Thinkers Corner, Enugu, Nigeria; ^2^Industrial Chemistry, Chemical Sciences Department, Godfrey Okoye University, PMB 01014, Thinkers Corner, Enugu, Nigeria

## Abstract

The global increase in oxidative stress related diseases such as cancer, cardiovascular, and inflammatory diseases caused by overwhelming level of free radicals in the body has encouraged the search for new antioxidant agents. Based on the ability of newly synthesized phenothiazine derivatives (6-chloro-11-azabenzo[a]phenothiazine-5-one and 6-[4-bromophenyl]-10-methyl-11-azabenzo[a]phenothiazine-5-one) to oxidize H_2_O_2_, a known free radical to sulfoxide, this study assessed the in vitro and in vivo antioxidant activity. The synthesized phenothiazine derivatives exhibited reducing power potential to convert Fe^3+^ to Fe^2+^ and high ability to scavenge H_2_O_2_ free radical in vitro. These activities were comparable to ascorbic acid, a standard antioxidant. The catalase activity significantly increased (*p* < 0.05) in groups 1 and 2 animals that received the phenothiazine derivatives compared to the controls (groups 3 and 4) suggesting the ability of the phenothiazine derivatives to scavenge H_2_O_2_ in vivo. The malondialdehyde level in groups 1 and 2 animals was lower than that in group 3 that received the reference compound (ascorbic acid) and group 4 that received the solvent suggesting the ability of the phenothiazine derivatives to prevent lipid membrane damage. AST and bilirubin levels were higher in group 2 animals which received 6-[4-bromophenyl]-10-methyl-11-azabenzo[a]phenothiazine-5-one compared to group 3, the positive control. The results suggest that phenothiazine derivatives, especially 6-chloro-11-azabenzo[a]phenothiazine-5-one, possess antioxidant activity though 6-[4-bromophenyl]-10-methyl-11-azabenzo[a]phenothiazine-5-one was slightly toxic. This activity may be due to the presence of electron donors such as sulfur as well as the richness of hydrogen in the additional benzene rings for substitution. Further study is needed to identify tolerable doses for possible therapeutic purposes.

## 1. Introduction

Based on man's activities and nature, we are constantly exposed to environmental pollutants, or other mechanical and chemical substances, solar radiation, and air for respiration which are capable of inducing the generation of free radicals or reactive oxygen species (ROS) [1]. Chemical species such as hydroxyl (OH^•^), superoxide (O_2_^•−^), nitric oxide (NO^•^), thyl (RS^•^), and peroxyl (RO_2_^•^), which contain unpaired electrons, are generally considered as free radicals. More so, certain chemical substances such as peroxynitrite (ONOO^−^), hypochlorous acid (HOCl), hydrogen peroxide (H_2_O_2_), singlet oxygen (^1^O_2_), and ozone (O_3_) which are not free radicals can easily lead to free radical reactions in living organisms [2–4]. During cellular processes such as respiration, certain reactive oxygen species are formed. If not removed, these free radicals which are negatively charged may attack positively charged centers in the cell and damage them. This damage may involve DNA and protein content of the cells and also lipid peroxidation of cellular membranes, calcium influx, and mitochondrial swelling and lysis thus promoting cellular injury and tissue damage [5, 6].

Naturally, the human body is adapted to maintain a condition of homeostasis by putting in place certain cellular defense systems to counteract the effect of these ROS or prooxidants. The strategies include prevention of damage, repair mechanism to alleviate the oxidative damages, physical protection mechanism against damage, and most importantly the antioxidant defense mechanisms to remove the prooxidants [7]. The endogenous antioxidant defense system includes both enzymatic and nonenzymatic antioxidant molecules that are usually distributed within the cytoplasm and various cell organelles [8].

Antioxidant enzymes, such as superoxide dismutase (SOD), catalase, and several peroxidases, catalyze a complex cascade of reactions to convert ROS to more stable molecules [9–11]. SODs catalyze the breakdown of O_2_^•−^ into O_2_ and H_2_O_2_ [11] while glutathione peroxidase and catalase catalyze the decomposition of H_2_O_2_ to H_2_O and O_2_ [12, 13]. Besides the primary antioxidant enzymes, a large number of secondary small molecular weight antioxidants molecules such as glutathione (GSH), NADPH, thioredoxin, albumin, transferring, metallothionein, uric acid, lipoic acid, ubiquinol, and trace metals, such as selenium act in close association with them as cofactors or coenzymes or direct scavengers to form redox cycles to maintain a delicate intracellular redox balance and minimize undesirable cellular damage caused by ROS [14, 15]. Other exogenous antioxidants from plants including vitamins E and C, carotenoids, and flavonoids also support the endogenous antioxidant defense system to eliminate free radicals [16].

In situations where the free radicals or oxidants level exceeds the natural antioxidant defense mechanism, a condition known as oxidative stress arises. Oxidative stress, defined by the imbalance between ROS level and the activity of the antioxidant defense in favor of the ROS, is an unhealthy condition which if severe can cause cell damage and lead to aging, several diseases conditions, or possibly death [17].

Today, oxidative stress is becoming a major global concern. The global trend of oxidative stress related diseases such as cardiovascular diseases, cancer, inflammatory diseases, ischemic diseases, acquired immunodeficiency syndrome, hypertension, and neurological disorders [18–20] are on the rise with an increase in mortality. Also, some metabolic diseases like diabetes which are also associated with an enhanced level of lipid peroxidation are equally increasing [21]. Managing such diseases entails supplementary antioxidants, to support the endogenous antioxidant defense system. Some natural exogenous antioxidants substances such as vitamins E and C, carotenoid, and flavonoid, which are readily consumed in food stuff, have been useful in such conditions [22, 23] but are, however, not usually sufficient to overcome the prooxidant level, especially when these foods are not consumed on a regular basis. Based on the chemistry of free radicals, chemically synthesized molecules with redox potentials which can be made available in required amount or dose are possible alternative to the natural occurring antioxidants.

Phenothiazine belongs to a class of heterocyclic compounds characterized by tricyclic aromatic ring with sulfur and nitrogen atoms. The heterocyclic nature with the presence of sulfur and nitrogen makes them a suitable pharmacological compound with a broad spectrum of biological activities. The first phenothiazine derivatives agents were successfully used for the treatment of psychosis [24]. Since then, these compounds have been of great pharmacological importance as they have been shown to possess various biological activities including antibacterial, antiviral, anti-inflammatory, and anticancer activities [25–27]. More so, phenothiazine structural motif has successfully been used in the design of a variety of pharmaceuticals which are clinically important for antioxidants activity, antitubercular activity, cholinesterase inhibitor, histamine H_1_ antagonist, and multiple drug resistance (MDR) reverting agent [28–31]. Substitution of phenothiazines ring has a great influence on their chemical properties. Because of the widespread application, synthesis and biological activity evaluation of phenothiazine and their derivatives have been subject to intense investigation [27].

In our previous works, we synthesized new tetracyclic and pentacyclic nonlinear phenothiazine derivatives: 6-chloro-11-azabenzo[a]phenothiazine-5-one [32] and 6-[4-bromophenyl]-10-methyl-11-azabenzo[a]phenothiazine-5-one [33], respectively. The ease of oxidation of these molecules with H_2_O_2_, a known ROS to sulphoxide, makes them suitable antioxidants. Thus, the present study sought to investigate these new phenothiazine derivatives for possible in vitro as well as in vivo antioxidant activity.

## 2. Materials and Methods

### 2.1. Phenothiazine Derivatives

**Figure 1:**
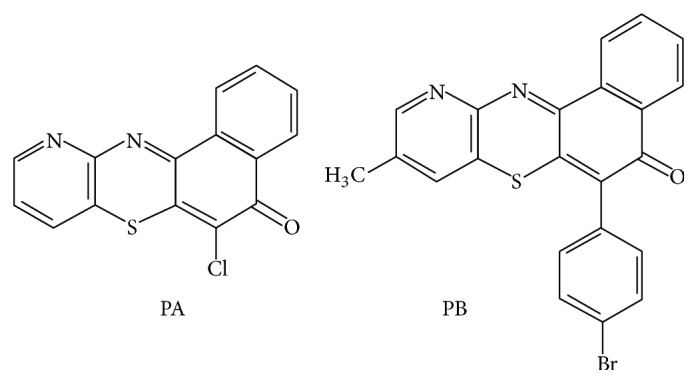
Phenothiazine derivatives. PA: 6-chloro-11-azabenzo[a]phenothiazine-5-one; PB: 6-[4-bromophenyl]-10-methyl-11-azabenzo[a]phenothiazine-5-one.

This study is a continuation of our previous study on the synthesis of new tetracyclic and pentacyclic nonlinear phenothiazine derivatives [28, 29]. The synthesized phenothiazine derivatives, 6-chloro-11-azabenzo[a]phenothiazine-5-one and 6-[4-bromophenyl]-10-methyl-11-azabenzo[a]phenothiazine-5-one, were transported from the Chemistry Laboratory of University of Nigeria Nsukka to the Chemistry Laboratory of Godfrey Okoye University, Enugu, Nigeria (Figure 1).

### 2.2. Preparation of the Compounds

The 6-chloro-11-azabenzo[a]phenothiazine-5-one compound was prepared by dissolving 330 mg of the compound in 10 mL of methanol, and 5 mL of benzene was added to make it up to 15 mL at a concentration of 22 mg/mL.

The 6-[4-bromophenyl]-10-methyl-11-azabenzo[a]phenothiazine-5-one compound was prepared by dissolving 141.3 mg in 13 mL of methanol, and 2 mL of benzene was added to make it up to 15 mL at a concentration of 9.42 mg/mL.

The solutions were stored at room temperature prior to the antioxidant assays.

### 2.3. In Vitro Antioxidant Activity

#### 2.3.1. Determination of Reducing Power

The reducing power of 6-chloro-11-azabenzo[a]phenothiazine-5-one and 6-[4-bromophenyl]-10-methyl-11-azabenzo[a]phenothiazine-5-one was determined following the method of Yen and Chen [34]. A volume of 1.0 mL of the phenothiazine derivatives and vitamin C at concentrations (0.125, 0.25, 0.5, and 1.0 mg/mL) was mixed individually with a mixture containing 2.5 mL of 0.2 M phosphate buffer (pH 6.6) and 2.5 mL of potassium ferricyanide (K_3_Fe(CN)_6_) (1% W/V). The resulting mixture was incubated at 50°C for 20 min, followed by the addition of 2.5 mL of trichloroacetic acid (10% W/V), and was then centrifuged at 3000 rpm for 10 min. A volume of 2.5 mL of the upper layer of the solution was mixed with 2.5 mL of distilled water and 0.5 mL of ferrous chloride (0.1%, W/V). The absorbance was measured at 700 nm against a blank sample. Increase absorbance of the reaction mixture indicated higher reducing power of the derivatives.

#### 2.3.2. Hydrogen Peroxide Scavenging Activity

The method used for the determination of the scavenging activity of H_2_O_2_ by the phenothiazine derivatives was according to Ruch and collaborators [35]. A volume of 4 mL of each of the phenothiazine derivatives and vitamin C at various concentrations (0.125, 0.25, 0.5, and 1.0 mg/mL) was mixed with 0.6 mL of 4 mM H_2_O_2_ solution prepared in phosphate buffer (0.1 M, pH 7.4) and incubated for 10 min. The absorbance of the solutions was taken at 230 nm against blank solution containing the phenothiazine derivatives without H_2_O_2_. The amount of H_2_O_2_ radical inhibited by the extract was calculated using the following equation:

(1) H2O2  radical  scavenging  activity=Abscontrol−AbssampleAbscontrol×100,

where Abs_control_ is the absorbance of H_2_O_2_ radical + solvent (methanol + benzene); Abs_sample_ is the absorbance of H_2_O_2_ radical + phenothiazine derivatives or vitamin C.

### 2.4. Animals and Handling

A total of 20 healthy albino rats of both sexes weighing 85 g–210 g were collected from the Department of Biological Sciences, University of Nigeria Nsukka, and transported to the animal house of Godfrey Okoye University, Enugu, Nigeria. The animals were housed in steel cages and were acclimatized at room temperature for a period of five weeks under standard environmental conditions with a 12-hour light/dark phase and were allowed access to food (top feeds, growers mash) and water ad libitum twice daily.

### 2.5. Experimental Design

The 20 rats were randomized into four groups consisting of five animals each and orally given treatment daily for seven days.

Group 1 were administered a solution of 6-chloro-11-azabenzo[a]phenothiazine-5-one at a dose of 50 mg/kg of body weight of the animal.

Group 2 were given a solution of 6-[4-bromophenyl]-10-methyl-11-azabenzo[a]phenothiazine at a dose of 25 mg/kg body weight of the animal.

Group 3 (positive control) received a solution of ascorbic acid at a dose of 90 mg/kg body weight of the animal.

**Figure 2:**
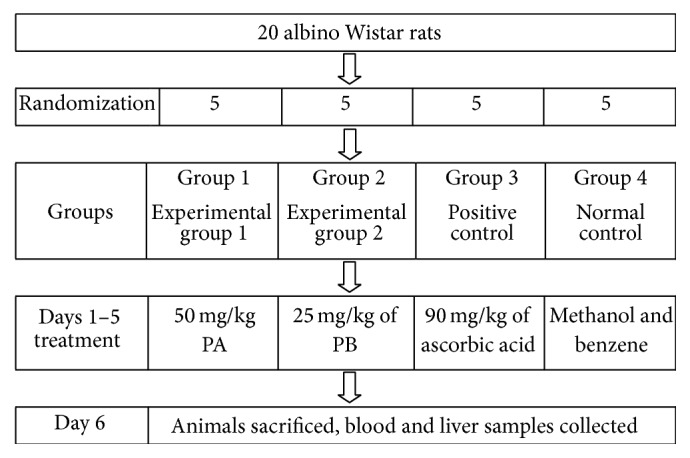
Experimental design chart. PA: 6-chloro-11-azabenzo[a]phenothiazine-5-one; PB: 6-[4-bromophenyl]-10-methyl-11-azabenzo[a]phenothiazine-5-one.

Group 4 which served as the normal control were given 0. 5 mL of a 2 : 1 mixture of benzene and methanol (experimental design is summarized in Figure 2).

### 2.6. Collection of Samples

After five days of treatment, the animals were fasted for 24 hours. Blood samples were collected from the different groups of animals via cardiac puncture and their livers were removed via dissection. The blood samples were centrifuged to obtain serum while the livers were washed with normal saline solution. The samples were stored at 4°C for further analysis.

### 2.7. In Vivo Antioxidant Activity

#### 2.7.1. Determination of Catalase Activity

The method described by Pari and Latha was employed for the determination of catalase activity [36]. The percentage of inhibition was evaluated following decrease in absorbance at 620 nm. The liver was homogenized in 0.01 M phosphate buffer (pH 7.0) and was centrifuged at 5000 rpm. The reaction mixture consisted of 0.4 mL of H_2_O_2_, (0.2 M), 1 mL of 0.01 M phosphate buffer (pH 7.0), and 0.1 mL of liver homogenate (10% w/v). Addition of 2 mL dichromate-acetic acid reagent (5% K_2_Cr_2_O_7_ prepared in glacial acetic acid) stopped the reaction. The absorbance was measured at 620 nm and recorded. The percentage inhibition was calculated using the following equation:

(2) Catalase%  inhibition=normal  activity−inhibited  activitynormal  activity×100,

where normal activity = hydrogen peroxide + phosphate buffer; inhibited activity = hydrogen peroxide + phosphate buffer + liver homogenate.

#### 2.7.2. Estimation of Lipid Peroxidation

The lipid peroxidation in the liver was measured using the modified method of Niehaus Jr. and Samuelsson [37]. It was measured colorimetrically by thiobarbituric acid reactive substances (TBARS). A volume of 0.1 mL of liver homogenate (10% w/v) was treated with 2 mL of (1 : 1 : 1 ratio) TBA-TCA-HCL reagent (thiobarbituric acid 0.37%, 15% trichloroacetic acid, and 0.25 N HCL). All the tubes were placed in a boiling bath for 30 minutes and cooled. The amount of malondialdehyde (MDA) formed in each of the samples was assessed by measuring the absorbance of clear supernatant at 535 nm against reference blank. Concentration of MDA was calculated using the equation

(3) $$\begin{array}{c} C=\frac{A}{E\times L}, \end{array}$$

where *A* is the absorbance of the sample, *E* is the extinction coefficient (1.56 × 10^5^ M^−1^ cm^−1^), and *L* is the length of the light path (1 cm).

### 2.8. Assessment of Liver Parameters

Aspartate aminotransferase (AST), alanine aminotransferase (ALT), and total bilirubin were assayed as prescribed by the Randox test kits, UK.

### 2.9. Statistical Analysis

The data obtained was analyzed using Statical Package for Social Sciences (SPSS) version 16.0 and the results expressed as mean ± standard error. Significant differences of the results were established by one-way ANOVA and the acceptance level of significance was *p* ≤ 0.05 for all the results.

## 3. Results and Discussion

Phenothiazine, also called “10*H*-phenothiazine,” is a nitrogen and sulfur containing electron-rich, tricyclic molecule. Phenothiazines have gained particular importance in pharmaceutical application [38]. Phenothiazines undergo reversible one-electron oxidation processes with low potentials which lead to stable and deeply coloured radical cations [39]. New phenothiazines derivatives have been shown to possess antioxidant activity [28]. This includes benzothiazines [40], azaphenothiazines, and phenothiazine-aryl amines conjugates via acetyl group [41, 42]. These phenothiazine derivatives have shown both in vitro and in vivo antioxidant activities.

**Figure 3:**
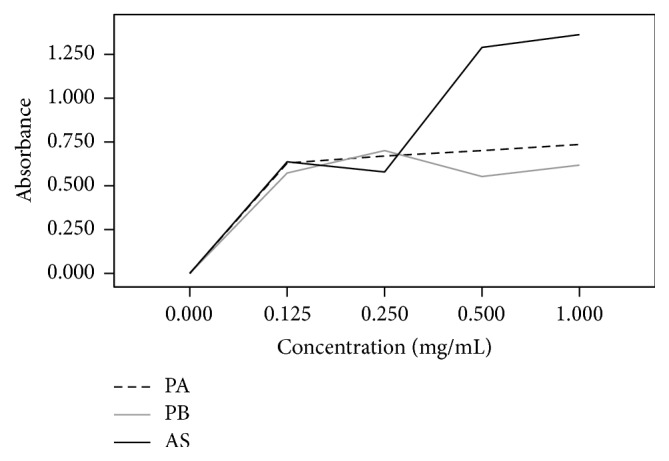
Reducing power activity of the phenothiazine derivatives compared to standard.

In this study, the in vivo and in vitro antioxidant potential of two newly synthesized phenothiazine derivatives was investigated. The reducing power of the phenothiazine derivatives was determined by measuring the transformation of Fe^3+^ to Fe^2+^. As shown in Figure 3, the phenothiazine derivatives exhibited redox potential to scavenge free radicals which were comparable to ascorbic acid, a standard antioxidant. The reducing power of the 0.125 and 0.25 mg/mL concentrations of both compounds was similar to that of the positive control (ascorbic acid). However, at higher concentrations of 0.5 and 1 mg/mL, the reducing power of the two synthesized compounds was lower than that of ascorbic acid. The reducing power of 6-chloro-11-azabenzo[a]phenothiazine-5-one was slightly higher than that of 6-[4-bromophenyl]-10-methyl-11-azabenzo[a]phenothiazine-5-one at concentrations of 0.5 and 1.0 mg/mL. The ability of these compounds to reduce Fe^3+^ to Fe^2+^ may be due to the free electrons present in sulfur in the phenothiazine ring.

As stated by Petrov and Van Breusegem, hydrogen peroxide is a toxic reactive oxygen species that induces damage to various biological molecules [43]. Hence, there is need for potential hydrogen peroxide reductants for possible elimination and prevention of cellular damage. As observed in this study, the phenothiazine derivatives showed high ability to scavenge hydrogen peroxide in the reaction mixture. Both compounds exhibited very high percentage inhibition of hydrogen peroxide (97.17 to 99.99%). Their activity was comparable to that of the reference compound (ascorbic acid) (Table 1). This is due to the presence of sulfur in their structures which reduces hydrogen peroxide to sulphoxide as suggested by previous studies [44, 45]. This ability of phenothiazine derivatives to inhibit hydrogen peroxide has been shown by* Maddila and collaborators as their newly synthesized* phenothiazine linked substituted benzylideneamino-1,2,4-triazole derivatives had the potentials to scavenge hydrogen peroxide [46].

**Figure 4:**
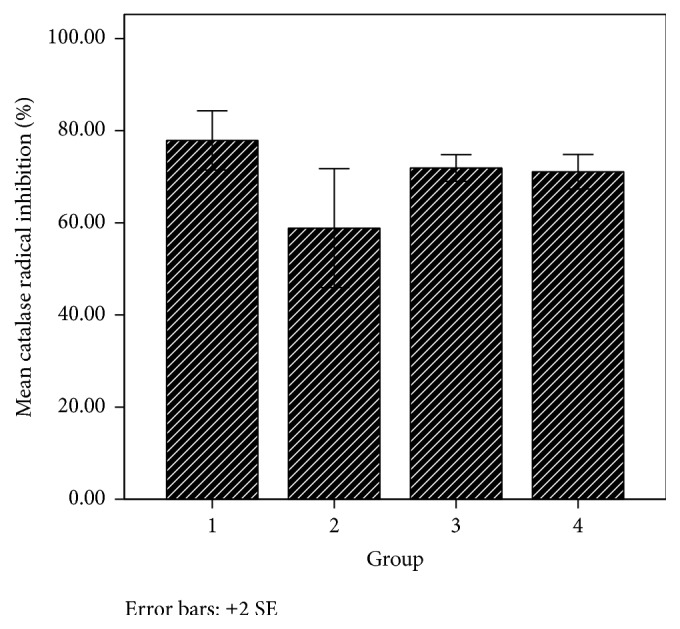
Catalase free radical scavenging potential in various animal groups. Group 1: 6-chloro-11-azabenzo[a]phenothiazine-5-one; group 2: 6-[4-bromophenyl]-10-methyl-11-azabenzo[a]phenothiazine-5-one; group 3: ascorbic acid; group 4: solvent.

Catalase is an enzyme present in living organisms including man that decomposes hydrogen peroxide into water and molecular oxygen, thereby protecting the tissues from highly reactive hydroxyl radicals [47]. In this study, catalase was shown to have an increase of percentage of inhibition of hydrogen peroxide in group 1 animals treated with 6-chloro-11-azabenzo[a]phenothiazine-5-one, group 2 animals given 6-[4-Bromophenyl]-10-methyl-11-azabenzo[a]phenothiazine-5-one, and group 3 given ascorbic acid (positive control) compared to group 4, the normal control, which was given organic solvents (Table 2 and Figure 4). The percentage of inhibition of hydrogen peroxide by catalase was significantly (*p* < 0.05) higher in group 1 animals given 6-chloro-11-azabenzo[a]phenothiazine-5-one than in those of group 2 given 6-[4-bromophenyl]-10-methyl-11-azabenzo[a]phenothiazine-5-one and insignificantly (*p* > 0.05) higher than those of group 3 which received ascorbic acid, the reference compound. This result suggests that 6-chloro-11-azabenzo[a]phenothiazine-5-one has high abilities to promote catalase activities in the body.

**Figure 5:**
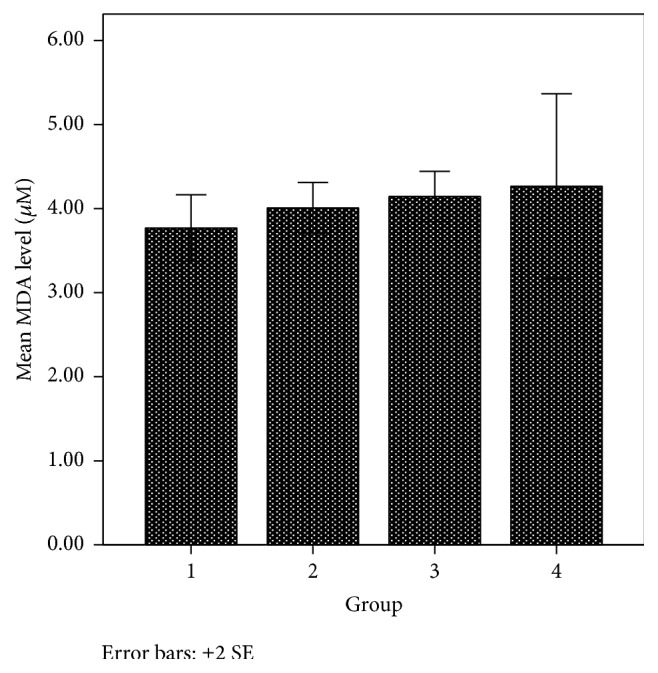
Malondialdehyde (MDA) level in various animal groups.

Malondialdehyde (MDA) is one of several low-molecular weight products formed via the decomposition of certain primary and secondary lipid peroxidation products during cell membrane damage [48]. At low pH and elevated temperature, MDA reacts with 2-thiobartituric acid (TBA), generating a red fluorescent with 1 : 2 MDA : TBA adduct. The amount of MDA in groups 1 and 2 animals that received phenothiazine derivatives was lower than in group 3 that received the reference compound (ascorbic acid) and group 4 that received the solvent. The amount of MDA formed was lowest in group 1 animals given 6-chloro-11-azabenzo[a]phenothiazine-5-one (Figure 5). This result suggests that these phenothiazine compounds can prevent or minimize lipid peroxidation or cell damaged caused by free radicals. Similarly, tetracyclic NH-azaphenothiazines were shown to exhibit significant antioxidant activity to inhibit lipid peroxidation in vitro which was suggested to be due to substitution of H, Cl, and OCH_3_ on the benzene ring [49]. Thus, in this study, the decrease in in vivo lipidic peroxidation may be due to the additional benzene rings of the newly synthesized phenothiazine derivatives which promoted substitutions.

**Figure 6:**
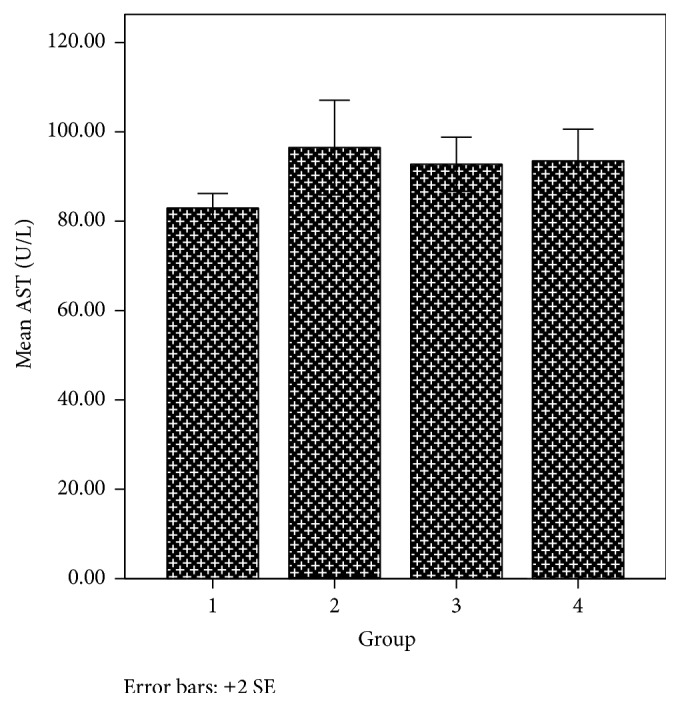
AST level in various animal groups.

**Figure 7:**
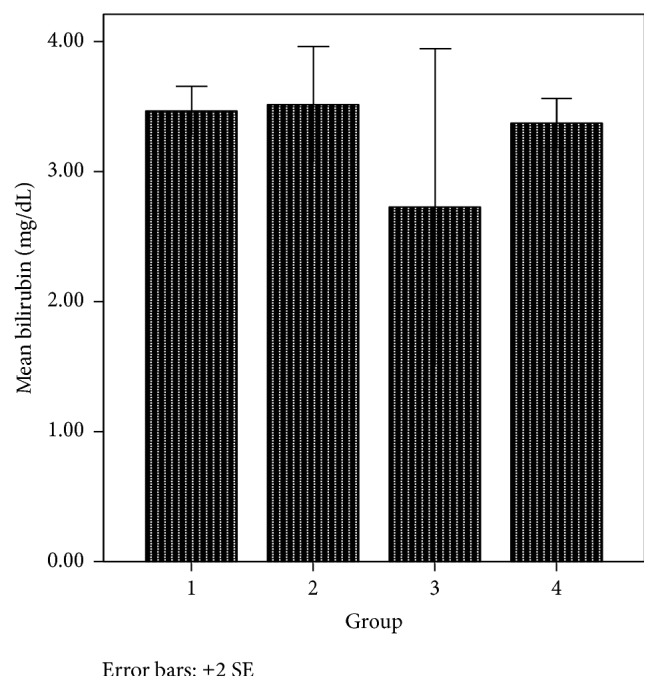
Bilirubin level in various animal groups.

**Figure 8:**
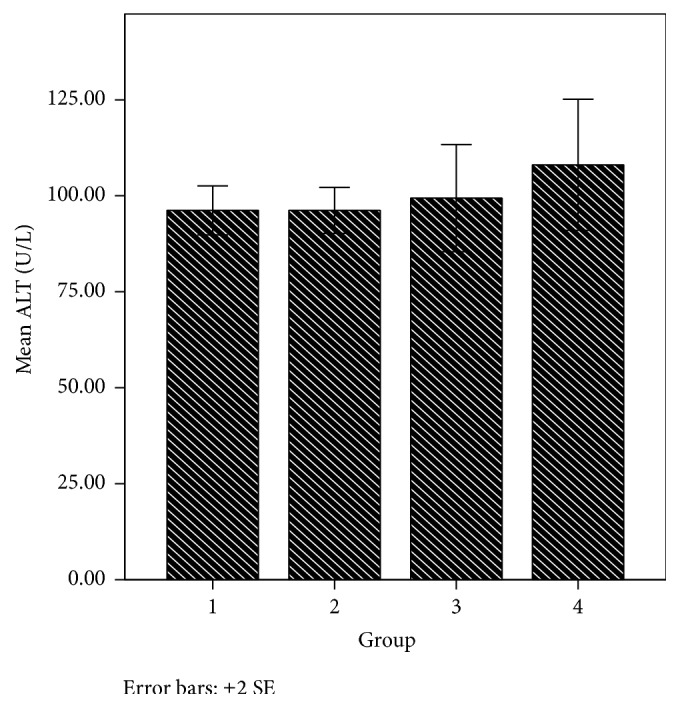
ALT level in various animal groups.

The liver is an organ in the body that metabolises drugs or xenobiotics to enhance their activity or facilitate their elimination. However, foreign substances may have adverse effect on the liver. Chemicals that cause liver injury are called hepatotoxins or hepatotoxicants. Hepatotoxicants are exogenous compounds of clinical relevance and may include overdoses of certain medicinal drugs, industrial chemicals, natural chemicals like microcystins, herbal remedies, and dietary supplements [50, 51]. Based on the fact that the newly synthesized phenothiazine compounds are of clinical relevance and are exogenous to the body, it was necessary to evaluate their effect on the liver. Biochemical markers like alanine aminotransferase [ALT], aspartate aminotransferase [AST], alkaline phosphatase [ALP], and bilirubin have been used to assess hepatotoxicity. Elevations in the serum enzyme levels of these markers are taken as the relevant indicators of liver toxicity. From the result obtained, the AST and bilirubin levels in group 2 animals treated with 6-(4-bromophenyl)-10-methyl-11-azabenzo[a]phenothiazine-5-one were slightly higher than that of groups 3 and 1 animals that received the reference compound and 6-chloro-11-azabenzo[a]phenothiazine-5-one, respectively, and AST increase was significant (*p* < 0.05) compared to the controls (Figures 6 and 7). However, the ALT levels did not significantly (*p* > 0.05) vary in the various animal groups (Figure 8). More so, more casualties were observed in the animal group that received 6-(4-bromophenyl)-10-methyl-11-azabenzo[a]phenothiazine-5-one. This suggests that these phenothiazine derivatives, most especially 6-(4-bromophenyl)-10-methyl-11-azabenzo[a]phenothiazine-5-one, may be toxic to the body at the given administered doses.

## 4. Conclusion

The newly synthesized tetracyclic and pentacyclic nonlinear phenothiazine derivatives, 6-chloro-11-azabenzo[a]phenothiazine-5-one and 6-(4-bromophenyl)-10-methyl-11-azabenzo[a]phenothiazine-5-one, possess potential in vitro and in vivo antioxidant activity. The overall antioxidant activity of these compounds may be due to the presence of electron donors such as sulfur as well as the richness of hydrogen in the benzene ring for substitution. However, though these compounds were slightly toxic to the animals, toxicity is dose dependent; thus further studies may be needed to identify the tolerable doses of these compounds with minimal adverse effect for possible applications. The antioxidant activity may be of potential useful therapeutic purposes to prevent or manage oxidative stress related diseases.

## Figures and Tables

**Table 1:** Hydrogen peroxide scavenging activity of phenothiazine derivatives.

Concentrations (mg/mL)	Percentage inhibition of H_2_O_2_ (%)		
	PA	PB	AS
1.000	99.42	99.99	99.55
0.500	99.99	99.99	99.88
0.250	97.71	98.80	99.96
0.125	97.47	99.99	99.99

**Table 2:** In vivo antioxidant activity ad liver parameters expressed in mean ± SE.

Group	1	2	3	4	*p* value
Catalase (% inhibition)	77.89 ± 3.26^a^	58.86 ± 6.46	71.90 ± 1.45	71.09 ± 1.87	0.016
Malondialdehyde (MDA) (*µ*M)	3.77 ± 0.20	4.01 ± 0.15	4.15 ± 0.15	4.27 ± 0.55	0.583
ALT (U/L)	96.20 ± 3.19	96.20 ± 3.00	99.40 ± 6.98	108 ± 8.55	0.605
AST (U/L)	82.95 ± 1.63	96.50 ± 5.30^a^	92.76 ± 3.04	93.53 ± 3.54	0.062
Bilirubin (mg/dL)	3.47 ± 0.09	3.52 ± 0.22	2.73 ± 0.61	3.37 ± 0.09	0.504

a superscript implies significant difference with group 4 (normal control).
